# In vivo NMR as a tool for probing molecular structure and dynamics in intact *Chlamydomonas reinhardtii* cells

**DOI:** 10.1007/s11120-017-0412-9

**Published:** 2017-06-23

**Authors:** Fatemeh Azadi-Chegeni, Christo Schiphorst, Anjali Pandit

**Affiliations:** 1 Department of Solid State NMR, Leiden Institute of Chemistry, Leiden University, Einsteinweg 55, 2333CC Leiden, The Netherlands; 2 Present Address: Department of Biotechnology, University of Verona, Verona, Italy

**Keywords:** Solid-state NMR, Polarization transfer, Conformational dynamics, Thylakoid membrane

## Abstract

We report the application of NMR dynamic spectral editing for probing the structure and dynamics of molecular constituents in fresh, intact cells and in freshly prepared thylakoid membranes of *Chlamydomonas reinhardtii* (*Cr*.) green algae. For isotope labeling, wild-type *Cr*. cells were grown on ^13^C acetate-enriched minimal medium. 1D ^13^C *J*-coupling based and dipolar-based MAS NMR spectra were applied to distinguish ^13^C resonances of different molecular components. 1D spectra were recorded over a physiological temperature range, and whole-cell spectra were compared to those taken from thylakoid membranes, evaluating their composition and dynamics. A theoretical model for NMR polarization transfer was used to simulate the relative intensities of direct, *J-*coupling, and dipolar-based polarization from which the degree of lipid segmental order and rotational dynamics of the lipid acyl chains were estimated. We observe that thylakoid lipid signals dominate the lipid spectral profile of whole algae cells, demonstrating that with our novel method, thylakoid membrane characteristics can be detected with atomistic precision inside intact photosynthetic cells. The experimental procedure is rapid and applicable to fresh cell cultures, and could be used as an original approach for detecting chemical profiles, and molecular structure and dynamics of photosynthetic membranes in vivo in functional states.

## Introduction

The plasticity of oxygenic photosynthetic membranes is tightly connected with plant fitness in fluctuating environments and their capability to respond to stress in excess light or drought conditions. Regulation of photosynthetic light harvesting is controlled by flexibility of the light-harvesting antenna from atomistic to supra molecular scale. Short- and long-term adaptation results in structural, dynamical changes varying from atomic-scale pigment and protein alterations to mesoscopic membrane rearrangements (Tardy and Havaux 1997; Cruz et al. 2005; Betterle et al. 2009; Erickson et al. 2015). Fast membrane remodeling is required to cope with sunlight fluctuations, while photosynthetic organisms may adjust their membrane compositions in adaptation to varying seasons or climates. The underlying regulation mechanisms have to be understood to the molecular level to gather central knowledge that can be used to increase plant stress tolerance or design algae species with improved solar-to-biomass conversion.

Essential here fore is the parallel development of suitable tools and methodology that can analyze molecular composition, structure, and plasticity of intact photosynthetic membranes of plants and cells grown under various environmental conditions or in different functional states. Fluorescence techniques have been developed for functional analysis of whole membranes, cells, or leaves, probing the dynamic nature of light harvesting in vivo in molecular detail (Lambrev et al. 2010; Amarnath et al. 2012; Ünlü et al. 2014; Wlodarczyk et al. 2015). Complementary techniques that can resolve conformational structures and dynamics to the molecular level inside physiological membranes or whole cells are still challenging. With Fourier-Transform Infra-Red (FTIR) spectroscopy, molecular information of protein carbonyls and lipids can be obtained from heterogeneous membranes, but has to be extracted from band fitting of broad FTIR absorbance spectra. The technique has been applied to determine the dynamics of protein and lipid moieties in *Synechocystis* cells and in higher-plant thylakoid membranes (Szalontai et al. 2000; Kóta et al. 2002). Resonance Raman (RR) spectroscopy can report on the conformations and H-bonding patterns of chromophores in intact systems and through this technique, it was discovered that light stress induces in vivo and in vitro changes in the conformation of neoxanthin (Neo) (Ruban et al. 2007). fluorescence recovery after photobleaching (FRAP) was used to investigate the lateral mobility of light-harvesting complexes in cyanobacteria and in plant thylakoid membranes (Mullineaux and Sarcina 2002). Thylakoid membrane fluidity has been investigated by measuring the rotational dynamics of externally added fluorescence or electron spin resonance (ESR) spin probes (Tardy and Havaux 1997). The latter techniques, however, do not report on the intrinsic, molecular dynamics of the membrane components. ^31^P NMR and fluorescence studies using fluorolipid probes have been applied to detect mesoscopic phase transitions in functional thylakoid membranes, and detected a transition from bilayer to inverted hexagonal states at high temperature (Krumova et al. 2008a, b). The advantage of ^31^P NMR is that no external probes are added and no isotope enrichment is required, and the disadvantage is however that only the phases of the phospholipids are followed, which in thylakoids only form ~10% of the total lipid composition. Recent multi-scale modeling simulations have provided molecular insight in thylakoid lipid lateral organization and dynamics (van Eerden et al. 2015) and predicted the molecular dynamics of Photosystem II embedded in a thylakoid membrane (Ogata et al. 2013; van Eerden et al. 2017). These studies have not been matched by experimental approaches, which would require detection of protein and lipid dynamics with atomistic resolution in native thylakoids, or atomic-level structural analysis of isolated pigment-protein complexes reconstituted in thylakoid lipid membranes.

**Fig. 1:**
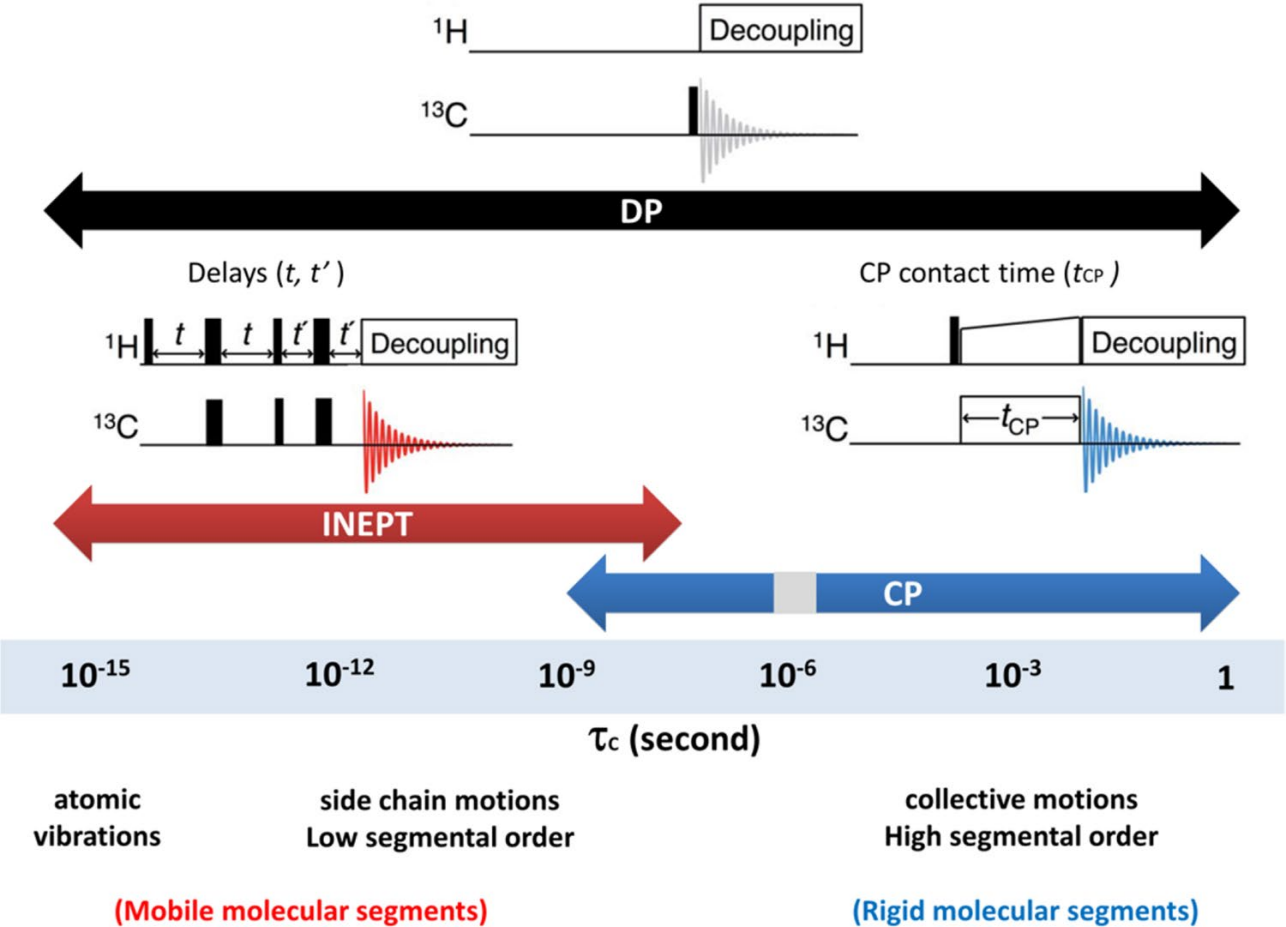
Schematic illustration of *DP, CP, INEPT* pulse sequences and dynamical ranges where the pulse experiments are effective

Herein, we describe the use of dynamic spectral-editing NMR as a new tool to analyze molecular composition and dynamics of thylakoid membranes or whole cells. Arnold et al. demonstrated that lipid and saccharide constituents could be identified in whole microalgae cells using NMR dynamic spectral editing to improve spectral resolution (Arnold et al. 2015). Topgaard and Sparr developed polarization-transfer solid-state NMR into a method that allowed them to detect molecular mobility in intact skin (Pham et al. 2017) and to determine surfactant phase transitions (Nowacka et al. 2010). In a recent study, we elaborated on these approaches to separate rigid and mobile thylakoid constituents in *Chlamydomonas reinhardtii* (*Cr*.) thylakoids (Azadi Chegeni et al. 2016) and demonstrated that protein and lipid molecules in zeaxanthin (Zea)-accumulating npq2 thylakoids display differential dynamics compared to WT (*cw15*) membranes, providing a molecular explanation for reported increased rigidity in Zea-rich membranes. The dynamic spectral-editing method, as explained in Azadi et al. (2016), consists of a combination of 1D ^13^C polarization-transfer solid-state NMR experiments. In ^1^H–^13^C polarization-transfer NMR, polarization is transferred from highly abundant nuclei ^1^H with high gyromagnetic ratio (*γ*) to low sensitive nuclei ^13^C with low *γ* and the resulting ^13^C spectrum is intensity enhanced, depending on the polarization-transfer efficiencies. The polarization transfer occurs via heteronuclear couplings between ^13^C and ^1^H nuclei, which are dipolar and scalar (*J*-) couplings. In CP-based experiments (Pines et al. 1972), polarization is transferred via dipolar couplings during a contact time *t*cp (Fig. 1, right pulse sequence). For mobile molecules, due to their fast random tumbling, dipolar couplings are zero-averaged within the contact time and consequently these components are filtered out from CP-based spectra. CP therefore acts as a dynamic filter that selectively probes rigid molecules. As shown in the “Results”, CP is also ineffective in a small dynamics range in the microseconds regime. In INEPT-based experiments (Morris and Freeman 1979) (Fig. 1, left pulse sequence), polarization is transferred via *J*-couplings that are not affected by bond re-orientation. INEPT is effective if the transverse (*T2*) relaxation times of the protons and carbons are sufficiently slow compared to the polarization transfer times. This is not the case for rigid molecules and therefore INEPT selectively probes mobile molecules (Nowacka et al. 2010). With direct polarization (DP) (Purusottam et al. 2013) (Fig. 1, upper pulse sequence), ^13^C are directly excited by applying a 90° pulse to the ^13^C carbons. DP in principal provides a ^13^C NMR spectrum of all molecular constituents.

A drawback of our previous study was the need for thylakoid extraction, which may alter membrane organization, and the fact that analyses were performed on extractions that were long-term stored and unfrozen for the experiments. In this paper, we demonstrate that our approach can be applied directly to fresh *Cr*. cells, circumventing isolation procedures and the need for sample storage. By simulating the polarization-transfer efficiencies, we show that lipid acyl-chain rotational dynamics and their degree of segmental order can be estimated within a certain range, in a quantitative way. Comparison of intact *Cr*. cells and freshly isolated thylakoid membranes show very similar NMR lipid spectral profiles, demonstrating that the thylakoid lipids dominate and implying that their molecular conformation and dynamics can be determined inside intact cells.

## Materials and methods

### Cell culturing

Wild-type *Cr*. cells (*strain cc124*) were grown mixotrophically on tris-acetate-phosphate (TAP) medium in a home-built set up, under continuous illumination with cool white LEDs (~50 μmol/m^2^ s) and constant temperature of 25 °C. For isotope-label incorporation, the acetic acid was replaced by ^13^C- acetate (Cambridge Isotopes).

### Pigment analysis

Chlorophyll and carotenoid concentrations were determined based on (Porra et al. 1989), using a home-written Javascript web application that performs a non-negative least-square fitting procedure based on Lawson and Hanson (1995) and Bro and de Jong (1997). In addition, pigment extracts were analyzed by High-performance liquid chromatography (HPLC).

### Thylakoid extraction

The isolation of thylakoids was performed according to Chua and Bennoun (1975) with some modifications. Cells were harvested in the exponential growth phase, centrifuged and resuspended in 0.2 volumes of MgCl_2_ buffer (1 mM MgCl_2_, 0.1 M HEPES, pH 7.5/KOH, 10% sucrose). Cells were ruptured by sonication on a 2500 W sonicator at 10%, using 15 cycles of 1 s on/10 s off followed by 30 cycles of 2 s on/10 s off. Thylakoid membranes were isolated using a discontinuous sucrose gradient. The disrupted cells were overlaid with 3 ml of 1.8 M sucrose in EDTA buffer, 1 ml of 1.3 M sucrose EDTA buffer, 1 ml of 0.5 M sucrose EDTA buffer and 5 ml of EDTA buffer without sucrose. The gradients were ultra-centrifuged for 1 h at 4 °C in a SW41 swing rotor (Beckmann) at 24.000 rpm (100.000×*g*).

### Solid-state NMR

NMR spectra were recorded on a Bruker Advance-III 750 MHz wide bore NMR spectrometer. NMR samples were prepared by mild centrifugation of fresh cell or thylakoid suspensions into a 4 mm NMR rotor that was used with a top insert. For cell samples, approximately 50 ml of cell culture was used and concentrated into the rotor, from which we estimate that samples contained about 0.5 mg of Chl. Magic Angle Spinning was performed at 5 kHz for whole cells and at 13 kHz for thylakoid extractions. Cross-polarization (CP) experiments were performed with a 2 ms CP contact time (*τ*_CP_), 5 s recycle delay and 20 ms acquisition time, *ω*_1_^*C*^/2*π* of 40.3 kHz and ^1^H nutation frequency linearly ramped from 80 to 100 kHz. Insensitive nucleus-enhanced polarization-transfer (INEPT) experiments were performed with two delays of 1.25 ms and an acquisition time of 80 ms. For direct polarization (DP) experiments, the delay was set at 5 s and the acquisition time was set to 43 ms. The dead time after pulse excitation before acquisition was 4.5 μs. Data were processed and analyzed in TopSpin3.2 and MNova. Temperature were calibrated analyzing ^207^Pb NMR chemical shifts of lead nitrate [Pb(No_3_)_2_] (Guan and Stark 2010).

### Simulation of INEPT and CP intensities

CP and INEPT intensities relative to DP as function of rotational correlation time *τ*_*c*_ and order parameter *S* were estimated using the following equations taken from (Nowacka et al. 2010, 2013):

1 $$\frac{{~I_{{{\text{CP}}}} }}{{I_{{{\text{DP}}}} }} = \frac{{\gamma _{{\text{H}}} }}{{\gamma _{{\text{C}}} }}~\frac{{\exp \left( { - {{\tau _{{{\text{CP}}}} } \mathord{\left/ {\vphantom {{\tau _{{{\text{CP}}}} } {T_{{1\rho }}^{{\text{H}}} }}} \right. \kern-\nulldelimiterspace} {T_{{1\rho }}^{{\text{H}}} }}} \right) - \exp \left( { - {{\tau _{{{\text{CP}}}} } \mathord{\left/ {\vphantom {{\tau _{{{\text{CP}}}} } {T_{{{\text{CH}}}} }}} \right. \kern-\nulldelimiterspace} {T_{{{\text{CH}}}} }}} \right)}}{{1 - {{\tau _{{{\text{CH}}}} } \mathord{\left/ {\vphantom {{\tau _{{{\text{CH}}}} } {T_{{1\rho }}^{{\text{H}}} }}} \right. \kern-\nulldelimiterspace} {T_{{1\rho }}^{{\text{H}}} }}}}$$

in which the gyromagnetic ratios of ^1^H and ^13^C are equivalent to 267.5 (10^6^ rad S^−1^ T^−1^) and 67.2 (10^6^ rad S^−1^ T^−1^), respectively, $T_{{1\rho }}^{\text{H}}$ is the ^1^H spin–lattice relaxation time in the rotating frame, *T*_CH_ is the time constant for cross-polarization, and *τ*_CP_ is the contact time for cross-polarization.

2 $$\frac{{I_{{{\text{INEPT}}}} }}{{I_{{{\text{DP}}}} }} = \frac{{\gamma _{{\text{H}}} }}{{\gamma _{{\text{C}}} }}n\sin \left( {2\pi J_{{{\text{CH}}}} \tau } \right)\sin \left( {2\pi J_{{{\text{CH}}}} \tau ^{'} } \right)\cos ^{{n - 1}} \left( {2\pi J_{{{\text{CH}}}} \tau ^{'} } \right)\exp \left( { - \frac{{2\tau }}{{T_{2}^{{\text{H}}} }} - \frac{{2\tau ^{'} }}{{T_{2}^{{\text{C}}} }}} \right)$$

in which *n* is the bond multiplicity, ${J}_{\text{CH}}$ is the ^1^H–^13^C through bond scalar coupling constant, and $T_{2}^{\text{H}}$ and $T_{2}^{\text{C}}$ are the effective ^1^H and ^13^C transverse dephasing times. The DP intensities in the equation are the theoretical intensities assuming that total polarization relaxation occurs after each pulse. The coherence evolution times *τ* = 1/4 *J*_CH_ and τ’ = 1/6 *J*_CH_ are delays between the radio frequency (rf) pulses in the INEPT sequence. $T_{2}^{\text{H}}$, $T_{2}^{\text{C}}$, $T_{{1\rho }}^{\text{H}}$ and *T*_CH_ values were estimated from a rotational correlation function that describes time-averaged fluctuations of the local magnetic field due to chemical-bond vector reorientations, depending on *τ*_c_ and *S* (see Nowacka et al. 2010, 2013). Experimental parameters that were used as input for the simulations were *τ*_CP_ = 2 ms, *J*_CH_ = 133.3 Hz, *ω*_*1*_^C^/2*π* = 40.3 kHz, *ω*_*1*_^C^/2*π* = 86 kHz, *ω*_*0*_^C^/2*π* = 188 MHz, *ω*_*0*_^H^/2*π* = 750 MHz, *τ*_*s*_ = 1 ms, *ω*_R_ = 5 kHz for cells and 13 kHz for isolated thylakoid membranes.

Curves of the relative INEPT intensities as function of rotational correlation time *τ*_*c*_ and order parameter *S* were generated using MathCad 15.0.

## Results and discussion

### Spectral editing and assignment of molecular constituents in* Cr.* intact cells

**Fig. 2:**
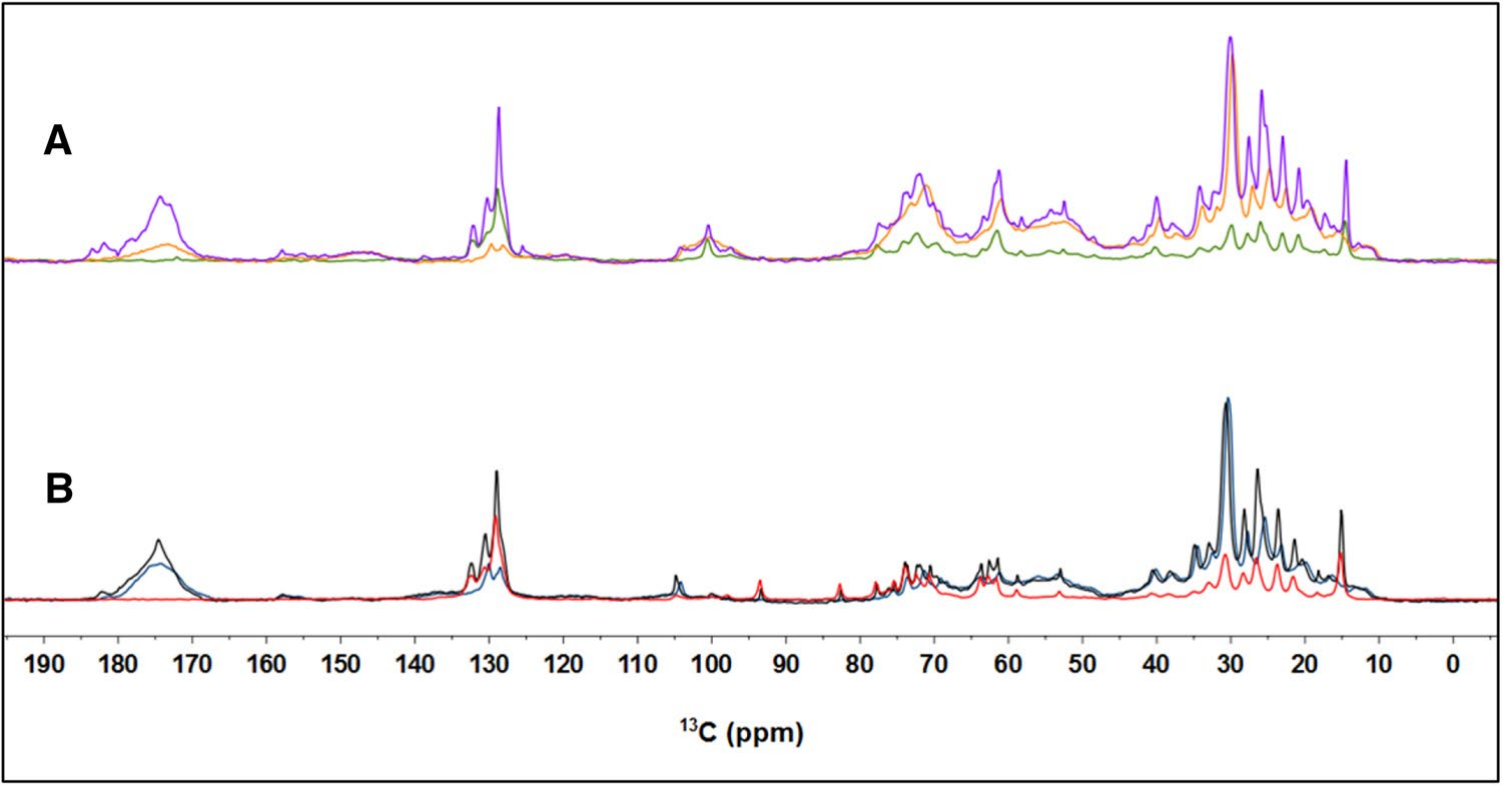
**a** Overlaid DP (*purple*), CP (*orange*) and INEPT (*green*) spectra of whole cells recorded at 13.5 °C. **b** Overlaid DP (*black*), CP (*blue*) and INEPT (*red*) spectra of isolated thylakoids recorded at 13.5 °C

**Fig. 3:**
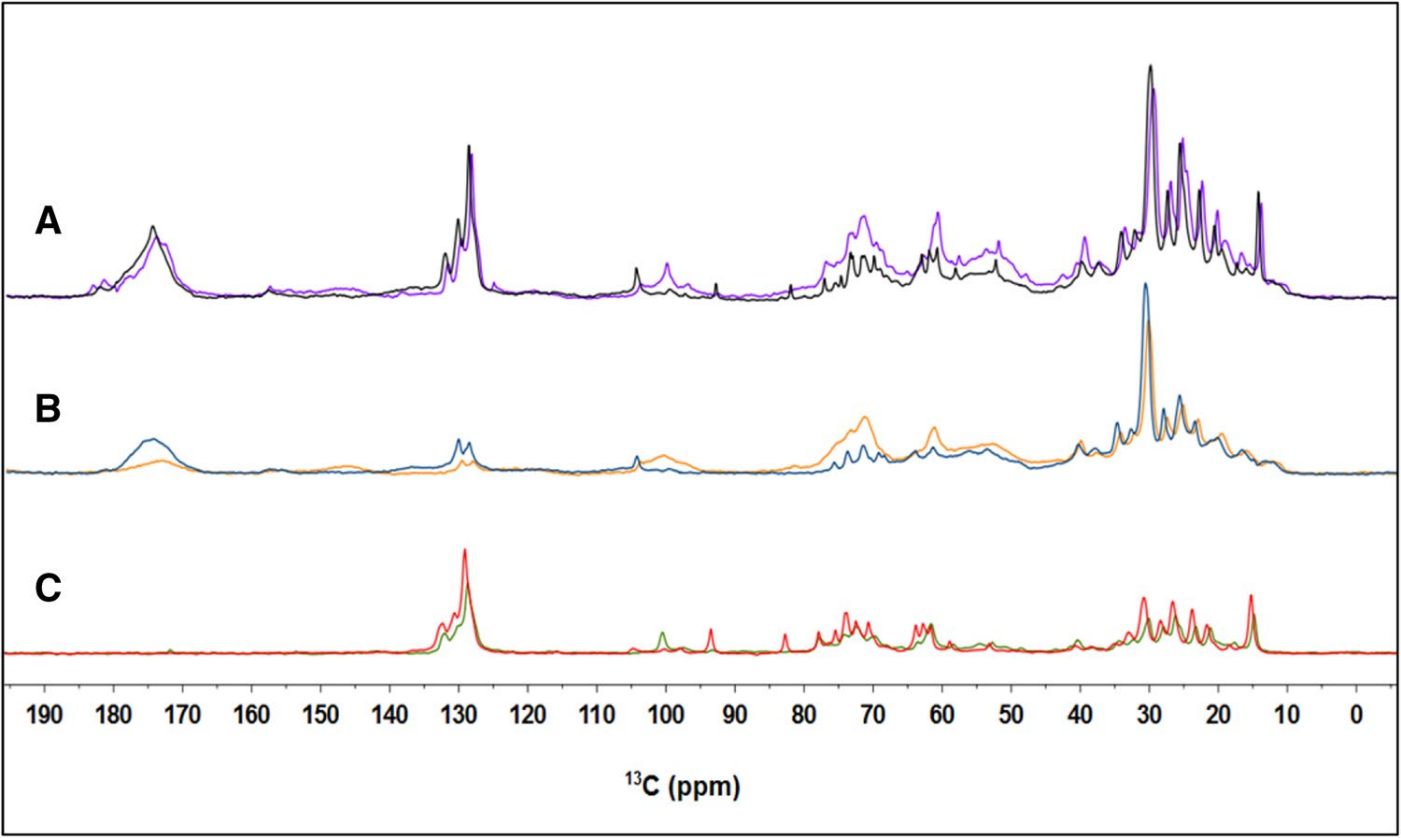
Overlaid DP, CP, and INEPT spectra of whole cells and thylakoids recorded at 13.5 °C. **a** DP spectra of whole cells (*purple*) and thylakoids (*black*). **b** CP spectra of whole cells (*orange*) and thylakoids (*blue*). **c** INEPT spectra of whole cells (*green*) and thylakoids (*red*)

Figure 2a presents a DP, CP and INEPT ^13^C spectrum of *Cr*. cells. We performed an assignment of the most prominent peaks in the ^13^C spectra. Most of the assignments are based on the following references (Moss 1976; Coddington et al. 1981; Lötjönen and Hynninen 1983; Castro et al. 2007; Arnold et al. 2015) and are summarized in Table 1. Many assignments are ambiguous and peaks correspond with reported NMR resonances of more than one possible carbon atom type, as indicated. *Cr*. lipid composition consists of monogalactosyldiacylglycerol (MGDG), digalactosyldiacylglycerol (DGDG), sulfoquinovosyldiacylglycerol (SQDG), diacylglyceryltrimethylhomo-Ser (DGTS), phosphatidylglycerol (PG) and phosphatidylethanolamine (PE) in descending order of abundance, and in case of overlapping peaks we only specify fatty acid (FA) chain length and degree of unsaturation, based on (Coddington et al. 1981). The region 10–40 ppm contains the protein side chain resonances that accumulate in a broad peak, together with the lipid FA CH_2_ and CH_3_ signals, which are the sharp peaks superimposed. The protein C_a_ signals are visible between 50 and 70 ppm and partly overlap with carbohydrate signals that are visible in the region 70–100 ppm. The region 125–135 ppm contains the signals of the aromatic protein side chains and double-bonded CH signals of the lipid FA. The protein backbone carbonyl signals accumulate between 170 and 180 ppm. Chl and xanthophyll signals are relative weak and coincide with other peaks. Figure 2 presents DP, CP and INEPT ^13^C spectra of *Cr*. whole cells (Fig. 2a) and of thylakoid membranes (Fig. 2b) and in Fig. 3 the spectrum of thylakoids and those of whole cells are superimposed for each type of NMR experiment. The lipid patterns in the aliphatic region (10–40 ppm) and aromatic region (125–135 ppm) are remarkably similar for both types of samples, which indicates that in whole cells, most of the lipids belong to the thylakoids. As expected, the signals from cell-wall components, starch or diacylglyceryl-*N*,/*V*,/*V*-trimethylhomoserine (DGTS) are absent in the spectra of thylakoids. The thylakoid membranes were purified with sucrose gradients, and signals in the region 70–100 ppm could originate from galactosyl lipid head groups but also from natural-abundant ^13^C of the sucrose present in the buffer. Between 135 and 140 ppm, a weak shoulder of the xanthophylls becomes visible in the DP and CP spectra of thylakoids. The protein carbonyl band has more intensity in the CP spectrum of thylakoids than in whole cells. This is because thylakoids contain membrane proteins that are relatively rigid and enhanced in CP, whereas intact cells contain a mixture of membrane and soluble proteins with higher mobility. The CH peaks of the unsaturated *ω3* fatty acids (FA) have much higher intensities in INEPT than in CP and the 132 ppm peak of the C16 carbons towards the FA end-tail are absent in CP, indicating that the majority of the* ω3* FA chains have low segmental ordering and highly dynamic FA tails.

**Table 1:** Chemical shift assignments

Chemical shift (ppm)	Assignment	INEPT	CP	References
12.6	Ile C_δ1_; xanth 9/13-Me		×	5, 6
14.6	Lipid CH_3_	×		1, 4
21.0	Lipid (*ω*-1) CH_2_ 18:3	×	×	1
23.2	Lipid (*ω*-1) CH_2_ 16:1/16:0	×	×	1
26.0	Lipid C3	×	×	1, 2
27.8	Lipid C8 18:3	×	×	1
30.2	Lipid CH_2_	×	×	4
32.5	Lipid C14 16:1/16:0	×	×	1
34.5	Lipid C2; Lut/Neo C1	×	×	1,5
37.6	Chl P7/11 phy; glycoprotein C3; xanth C1	×	×	3,5
40.1	Chl phytols P8/10/12; xanth C4	×	×	3,5
52.6	Glycoprotein C; PC C_γ_	×	×	2
61.7	Glyc C1/C3; glycoprotein C2	×	×	2,4
63.5	Glyc C1/C3	×		2,4
71.5	MGDG G3		×	2,4
72.5	Lipid G2/3/5, starch C2/5	×		2
73.6	Lipid G2/3		×	2,4
74.3	Starch C3	×	×	2
78.0	Glycoprotein C4	×		2
98.0	SQDG/DGDG G1	×	×	2,4
100.5	DGDG G1, starch C1	×	×	2
104.2	MGDG G1; cell-wall C1	×	×	2,4
128.6	Lipid C10 18:3	×	×	1
130.1	Lipid C9/12/13 18:3	×	×	1,5
132.0	Lipid C16 18:3	×		1,5
157.7	Arg C_ζ_			6
171.9	Lipid C1/CO	×		2
172.6	DGTS C1/CO			2
174.2	PG C1			2
181.7	Glu/Asp COO^−^		×	6
183.1	Glu/Asp COO^−^		×	6

*Crosses* indicate that the peak is observed in the ^13^C INEPT, resp. CP spectrum

*Xanth* xanthophyll, *Phy* Chl phytol chain, *Lut* lutein, *Neo* neoxanthin, *Glyc* glycerol

*1* Coddington et al. (1981), *2* Arnold et al. (2015) and references therein, *3* Lötjönen and Hynninen (1983), *4* de Souza et al. (2007), *5* Moss (1976), *6* Ulrich et al. (2008)

### Temperature dependence

**Fig. 4:**
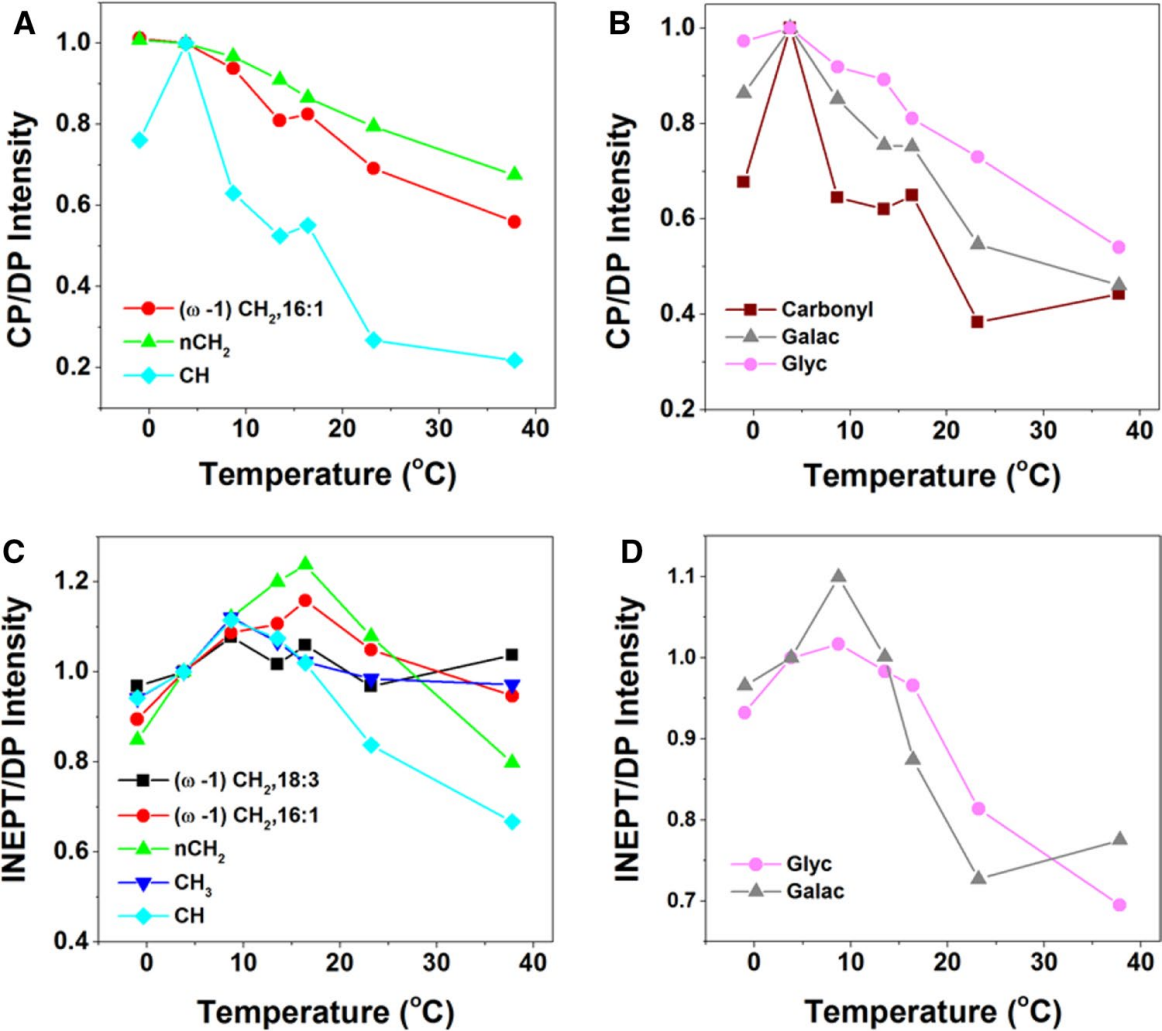
^13^C CP and INEPT intensities of whole-cell components as a function of temperature. The intensities are normalized at *T* = 3 °C. **a** CP intensities of fatty acids. *Red circles* (*ω*-1)CH_2_ of 16:1; *green triangles n*CH_2_; *light-blue squares* CH. **b** CP intensities of protein carbonyls and carbohydrates. *Dark-red squares* carbonyl; *gray triangles* galactosyl and glycerol carbons (Galac) of glycerolipids; *pink circles* glycoprotein and glycerol C1/C3 carbons (Glyc). **c** INEPT intensities of fatty acids. *Black squares* (*ω*-1)CH_2_ of 18:3; *red circles* (*ω*-1)CH_2_ of 16:1; *green triangles n*CH_2_; *dark-blue triangles* CH_3_; *light-blue squares* CH. **d** INEPT intensities of protein carbonyls and carbohydrates. *Gray triangles* galactosyl and glycerol carbons of glycerolipids; *pink circles* glycoprotein and glycerol C1/C3 carbons

**Fig. 5:**
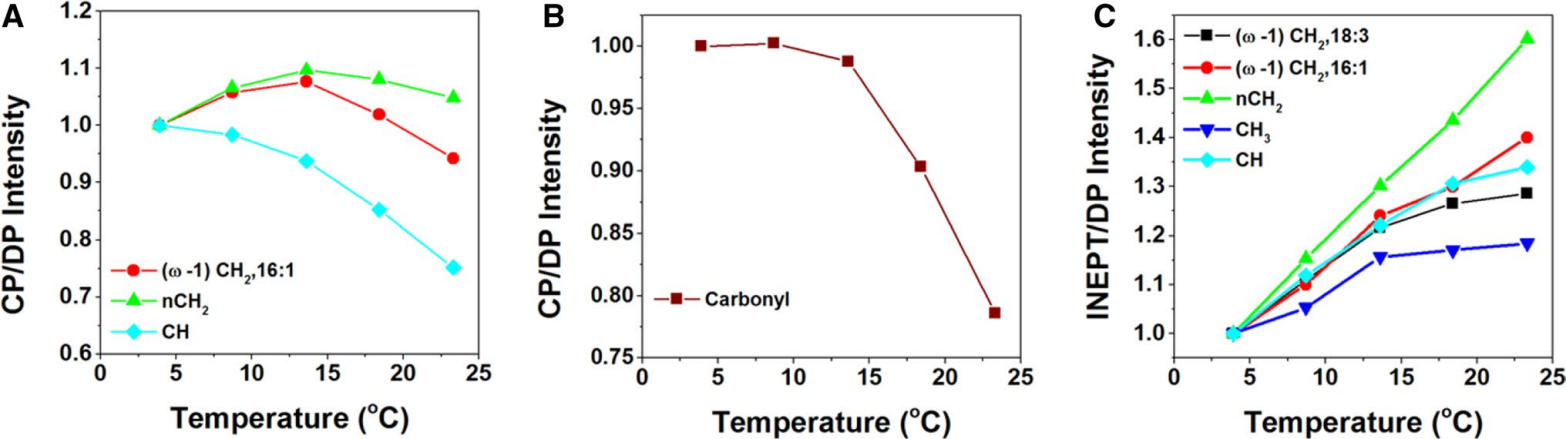
^13^C CP-MAS and INEPT intensities of thylakoids as a function of temperature. **a** CP intensities of fatty acidcs. *Red circles* (*ω*-1)CH_2_ of 16:1; *green triangles n*CH_2_; *light-blue squares* CH. **b** CP intensities of protein carbonyls. **c** INEPT intensities of fatty acids. *Black squares* (*ω*-1)CH_2_ of 18:3; *red circles* (*ω*-1)CH_2_ of 16:1; *green triangles* CH_2_; *dark-blue triangles* CH_3_; *light-blue squares* CH

The dynamic behavior of protein and lipid components was further explored by varying the temperature in a physiological range. For thylakoid membranes, the temperature was not raised above 23.3 °C to make sure that no irreversible damage would occur. For whole cells, the temperature was raised to 37.8 °C. To check if heating caused irreversible changes, the temperature was raised and lowered again after which samples were re-measured, verifying that no heating-induced changes had taken place (data not shown). We noticed a decrease of signal intensity in DP spectra over time, which could be caused by the effect of magic-angle spinning, slowly sedimenting the cells at the rotor walls and changing the filling factor. To correct for time-dependent intensity changes, the CP and INEPT intensities were divided by the DP intensities of the respective peaks. Figure 4 presents the temperature-dependent CP over DP and INEPT over DP signal intensities of whole-cell spectral components, and Fig. 5 presents the temperature curves obtained from thylakoid spectra. The intensities are normalized with respect to the intensity at 3 °C for better comparison. The thylakoid INEPT-observed components (Fig. 5c) show an increase with temperature that seems to stabilize for around 20 °C for the CH and FA end-tail carbons. The thylakoid CP-observed components (Fig. 5a, b) show a decrease of CP efficiencies with temperature except for the *n*CH_2_ carbons. Because INEPT signals are sensitive for highly mobile components and CP signals are enhanced for more rigid molecules, this behavior is in line with expected increase of mobility at higher temperatures. The whole-cell CP-observed components (Fig. 4a, b) also show an overall decrease of CP efficiencies with temperature, but unexpectedly the INEPT-observed intensities (Fig. 4c, d) only increase up to 13.5 °C and stabilize or decrease at higher temperatures, suggesting that their dynamics is reduced.

### Simulated INEPT and CP efficiencies

**Fig. 6:**
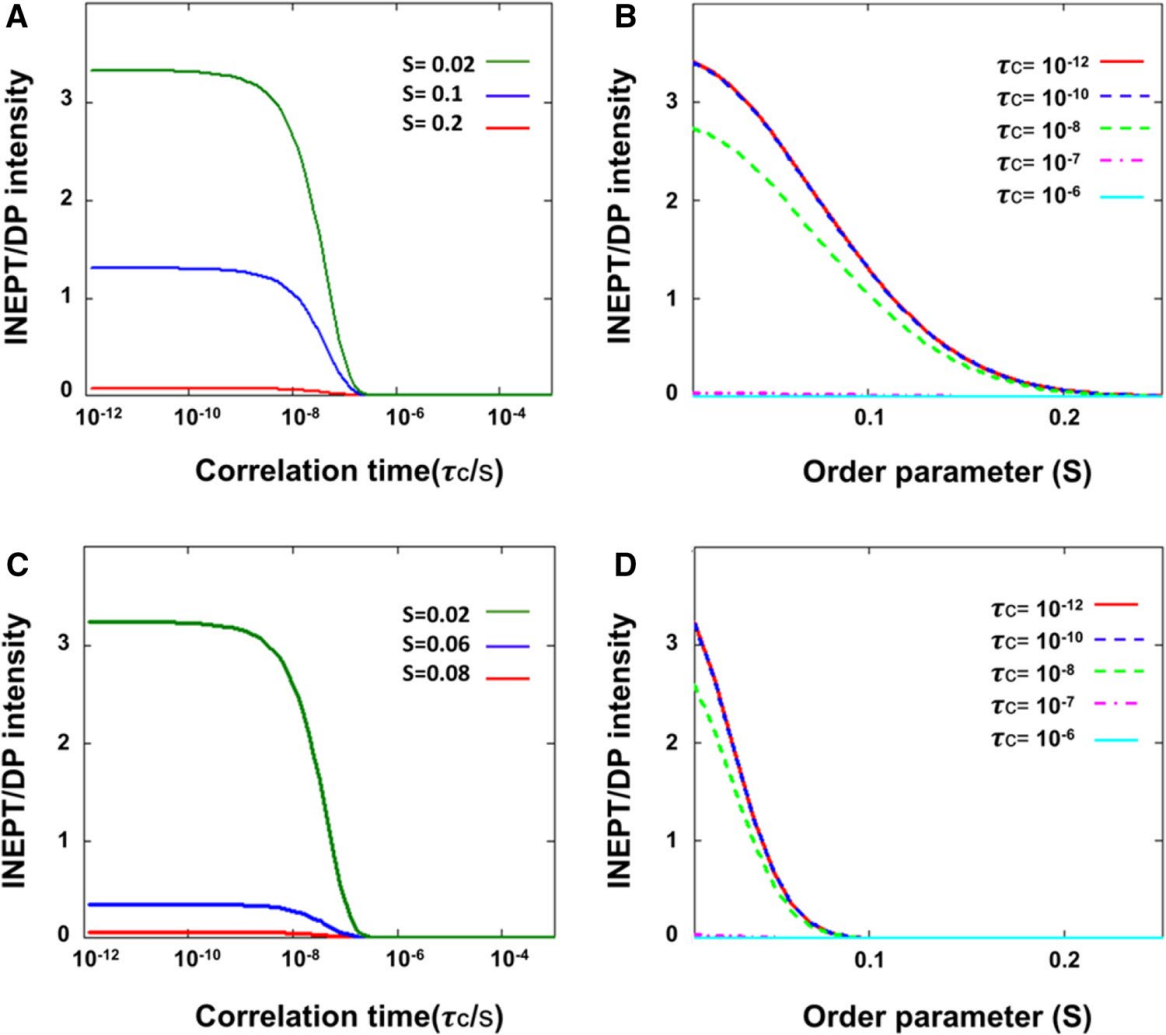
Simulated INEPT/DP intensities as a function of order parameter *S* and rotational correlation time *τ*_c_ for a CH segment. **a** INEPT/DP intensities for *ω*_R_ = 13 kHz (simulating the thylakoid experiments) as function of *τ*_C_ at *S* = 0.2, 0.1 and 0.02. **b** INEPT/DP intensities for *ω*_R_ = 13 kHz as a function of *S* at *τ*_C_ = 10^−12^, 10^−10^, 10^−8^, 10^−7^ and 10^−6^ s. **c** INEPT/DP intensities for *ω*_R_ = 5 kHz (simulating the whole-cell experiments) as a function of *τ*_C_ at *S* = 0.08, 0.06 and 0.02. **d** INEPT/DP intensities for *ω*_R_ = 5 kHz as a function of *S* at *τ*_C_ = 10^−12^, 10^−10^, 10^−8^, 10^−7^ and 10^−6^ s

**Fig. 7:**
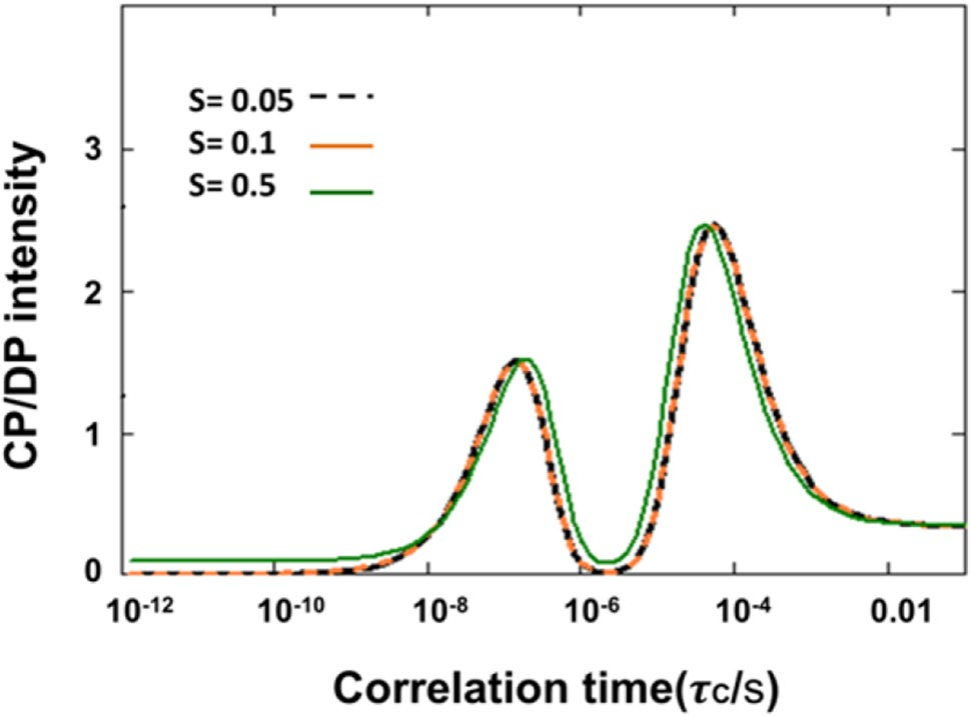
Simulated CP/DP intensities as a function of rotational correlation time *τ*_c_ for *S* = 0.05, 0.1 and 0.5

To gain more insight how the experimentally obtained INEPT and CP intensities related to molecular dynamics and segmental ordering, we simulated the INEPT and CP intensities as function of rotational correlation time *τ*_C_ and order parameter *S*, using our experimental NMR parameters as input. In Fig. 6, we show the theoretical INEPT polarization-transfer efficiency for a CH segment. Figure 6a, b present *I*_INEPT_/*I*_DP_ as a function of correlation time (*τ*_C_) and of order parameter (*S*) at MAS frequency of 13 kHz (simulating the thylakoid experiment), and Fig. 6c, d present *I*_INEPT_/*I*_DP_ calculated for a MAS frequency of 5 kHz (simulating the whole-cell experiment). At both MAS frequencies, INEPT starts to be effective for correlation times below 0.1 μs (*τ*_C_ < 0.1 μs) and approaches a maximum (*I*_max_) at *τ*_C_ < 1 ns. In the sub-nanosecond dynamics range, the INEPT intensities only depend on the segmental order parameter *S*. At 13 kHz MAS, INEPT becomes effective at *S* < 0.2, while at 5 kHZ, INEPT becomes effective at *S* < 0.05, and has a lower *I*_max_. We performed the same analysis for CH_2_ and CH_3_ segments and summarized the results in Table 2. Figure 7 presents the theoretical *I*_CP_/*I*_DP_ intensities as a function of correlation time for a CH segment. The predicted CP intensities were identical for 5 and 13 kHz. Figure 7 shows that CP is effective for correlation times between nanoseconds and seconds, except for a gap in the microsecond range in which CP gives no signal. The same behavior as function of the correlation time is observed for different order parameter values (*S* = 0.05, 0.1 and 0.5) except that for *S* ≥ 0.5, CP has non-zero intensity for sub-nanosecond *τ*_C_ values. Hence, CP is effective for oriented molecules with restricted motions, even if they exhibit very fast dynamics.

**Table 2:** Theoretical conditions for observing INEPT intensities at 5 kHz (simulating the whole-cell experiments) and 13 kHz (simulating the thylakoid experiments)

Spinning frequency (*ω*_R_)		Correlation time μs (*τ*_c_)	Order parameter (*S*)
5 kHz	CH	<0.1	<0.08
	CH_2_	<0.05	<0.05
	CH_3_	<0.05	<0.1
13 kHz	CH	<0.1	<0.2
	CH_2_	<0.05	<0.1
	CH_3_	<0.05	<0.03

### Dynamics of *Cr*. cell and thylakoid molecular components

We used the simulations to estimate the dynamics of *Cr*. cell constituents, in particular of the lipids, of which signals are observed both in INEPT and CP. The simulated intensity ratios use *I*_DP_ assuming that DP is effective over the whole frequency range. The experimentally observed DP intensities however depend on *T1* and on the delay time *t*_d_ between scans, so that *I*_DP_/*I*_DP theory_  = 1 − exp(−t_d_/$T_{1}^{\text{C}}$). With our experimental settings, we assume that DP intensities are attenuated for components with *τ*_C_ > 1 μs. Since the DP spectra show significant intensity for most of the cell and thylakoid components, we presume that these have dynamics in the sub-microsecond range. Comparing the spectral profiles of whole cells and thylakoids (Fig. 3), we conclude that the whole-cell INEPT spectrum is dominated by the signals of the thylakoid lipids. The higher intensities observed for the thylakoid INEPT spectrum compared to the whole-cell INEPT spectrum, relative to the DP intensities (Fig. 2a, b), can be explained by the increased MAS frequency used for the thylakoid experiments that renders INEPT more efficient, as illustrated in the model. The largest *n*CH_2_ lipid peak at 30 ppm has similar intensity in CP and DP for both whole cells and thylakoids (Fig. 2a, b), conforming to *I*_CP/DP_ ~ 1 in our model. Focusing on the sub-microsecond regime where DP is effective, *I*_CP/DP_ ~ 1 matches with *τ*_c_’*s* in the order of 50 ns. In contrast to the *n*CH_2_ carbons, the CH carbons have much larger signals in INEPT than in CP, implying that they have conformational dynamics with *τ*_c_ < 10 ns and a large degree of disorder (*S* < 0.05 for whole cells). The lipid CH carbons are the bended, lower part of the unsaturated FA chains, while the *n*CH_2_ FA carbons are located toward the lipid head groups. The lipid FA chains of the unsaturated lipids thus become more dynamic towards the end tails, which are disordered and undergo fast motions.

In the temperature profiles of the intact cells (Fig. 4), we observe overall a decay of the CP intensities for lipid (Fig. 4 a), and protein backbone and cell-wall (Fig. 4b) components at higher temperatures, indicating that the cell components have increased molecular motions. The lowest temperature point in the curves in Fig. 4 was close to zero Celsius, which could have led to non-linear temperature effects, and might explain the observed increase instead of decrease of CP signal between the two lowest temperature points. The INEPT-observed lipid signals (Fig. 4c) increase for temperatures up to ~13 °C, indicating that the lipid segments increase their conformational dynamics, or become more disordered. Above this temperature, the signals remain constant for the end-tail carbons, suggesting that their conformational dynamics enter the sub-nanosecond regime where INEPT becomes insensitive to further changes in dynamics, and that there is no change in lipid disorder. Interestingly, the INEPT-observed *n*CH_2,_ CH (Fig. 4c) and galactosyl/glycerol (Fig. 4d) signals clearly decline at higher temperatures, suggesting that conformational dynamics of mobile lipid head groups and FA segments are suppressed. The lipid spectral profiles do not change with temperature, and from analysis of lipid peak intensities, we exclude significant changes in lipid unsaturation or isomerization. The reduced lipid dynamics at high temperatures could be associated with a phase transition in the membrane. The INEPT temperature curves suggest that there is a mechanism (phase transition or other) that effectively protects the photosynthetic membranes against extreme membrane fluidity at high temperatures, unrelated to lipid saturation or isomerization, which would be of interest to further explore.

The temperature profiles of the thylakoid sample (Fig. 5) cannot directly be compared to the whole-cell sample, because of the increased sensitivity for INEPT detection in the thylakoid experiment with the higher MAS frequency used and because of the limited temperature range over which the thylakoid preparations could be recorded without thermal damage. In the overlapping temperature range, the overall dynamic behavior is similar. The thylakoid INEPT-observed lipid signals in this work (Fig. 5c) are higher than observed in the study of Azadi et al. (2016), where we concluded that the major lipid fraction did not display fast dynamics, while a smaller fraction had mobile FA tails. In that study, we also observed larger effect of lipid isomerization with temperature. Here, we observe only small effect of isomerization and find that the majority of the lipids have very flexible tails. A difference in sample conditions was that in the former study the water content of the rotor sample preparations was lower, which could have reduced the lipid conformational dynamics. In addition, the thylakoid preparations studied in Azadi et al. were from different strains and had been stored at −80 °C before use, using glycerol as a cryo-protectant, whereas the thylakoid preparation in this study contained sucrose and was freshly prepared.

### Evaluation of the method: limitations and possibilities

We demonstrate that ^13^C NMR spectral editing can identify lipid, protein, sugar, and cell-wall constituents and resolve and quantify molecular dynamics of different cellular components, in particular lipid FA, in intact photosynthetic cells. Sample preparation typically involved growing a 50 ml *Cr*. culture for 2–3 days and using 1,2-^13^C acetate as a carbon source, which is very feasible both in terms of labor and label expenses. The presented spectra were each recorded with 64 scans, which typically takes about 10 min for scan accumulation. However, we noticed that after rotor insertion, spectral changes occurred during MAS in the first 30–60 min, presumably due to slow sedimentation of cells on the rotor walls. Thus, the samples need to be spun for at least 1 h in the rotor at the set spinning frequency before starting the measurements. MAS spinning did not break the cells at 5 kHz spinning frequency, which was checked afterward using an ordinary light microscope.

The motivation for this study was to explore NMR-based methods that could eventually probe molecular structure and dynamics of photosynthetic membranes in vivo in functional cell systems. A clear advantage of the NMR approach is that, additional to dynamics information, full analytical profiles are obtained. This allows to the researcher to simultaneously have an overview of cell or membrane chemical compositions and conformations (lipid isomers, degree of unsaturation). Spectral-editing approaches have recently been combined with multiphase NMR, a novel technology that integrates hardware from solution-, gel-, and solid-state into a single NMR probe, and even permitted simultaneous identification or metabolites and molecular structure in a living organism (Mobarhan et al. 2016). For the purpose here of studying thylakoid membrane plasticity, a limitation is the lack of sensitivity for specific proteins (such as LHCII) or lipid types [such as MGDG which can induce membrane hexagonal phases (Krumova et al. 2008a)]. In that respect, FRAP is more selective for study of light-harvesting protein mobility, but has limited resolution since lateral diffusion is measured, while NMR detects rotational diffusion of molecules with atomistic resolution. We believe that our novel NMR approach can be further exploited by integration with selective mutation or labeling approaches. Distinction of different lipid types will be improved when cell-wall deficient algae strains are used, because the cell-wall components resonate in the region where the lipid galactosyl-head group signals occur. For instance, the MGDG G1 head-group carbons have distinctive chemical shifts at 104 ppm that differentiate them from DGDG, but that in our spectra overlap with signals from cell-wall components. Dinc et al. created a *Cr*. “minimal cell” depleted of Photosystem I and II core components (Dinc et al. 2016) that would perform non-photochemical quenching (NPQ) with only LHCII and LHCSR present as Chl-containing antenna proteins. Such minimal system could be used to more selectively probe the dynamic behavior of LHCII in a membrane or cellular environment under normal or quenched conditions. Selective ^13^C in vivo labeling of Chls could be achieved by addition of *δ*-aminolevulinic acid to the cell growth medium (Janssen et al. 2010), which would amplify the Chl signals with respect to the lipid and cellular background.
